# Application of sex/gender-specific medicine in healthcare

**DOI:** 10.4069/kjwhn.2023.03.13

**Published:** 2023-03-31

**Authors:** Nayoung Kim

**Affiliations:** Department of Internal Medicine, Seoul National University Bundang Hospital, Seongnam, Korea

## Introduction

The study of differences between men and women in medicine is referred to as “sex/gender-specific medicine” (SGM) and is crucial in medical practice. In 2021, I published the Korean version of *Sex/Gender-Specific Medicine in the Gastrointestinal Diseases* [1], and its English version [2] was published by Springer, a top-tier publisher, in 2022. This book has been recognized as a textbook that systematizes the concept of SGM and may be the first medical book related to SGM in South Korea (hereafter, Korea). When I first introduced the concept of SGM to acquaintances, I remember they searched Google with awkward facial expressions, thinking it referred to research on sexual minorities. Thus, SGM is not yet universally known in Korea.

My interest in SGM began in 2014 when I participated in the Gendered Innovation Workshop between Stanford University and the Korean Federation of Women’s Science and Technology Associations (KOFWST). While assisting Professor Mi-Kyung Sung from the Department of Food and Nutrition at Sookmyung Women’s University with her presentation on colorectal cancer from a clinical perspective, I became interested in SGM in colorectal cancer. Subsequently, in 2013, I began researching SGM earnestly as a participant in the Korean Center for Gendered Innovations for Science and Technology Research (GISTeR) established under KOFWST. In 2016, I was able to conduct balanced research on both basic and clinical SGM as a participant in the “Promoting Excellence and Practicability of Science and Technology Research through Gendered Innovation” project of the Ministry of Science, ICT and Future Planning. The “Sex and Gender Research in Medical Science” classes held in 2017 and 2019 at the Graduate School of Translational Medicine at Seoul National University College of Medicine received positive feedback from medical students. Later, *Sex/Gender-Specific Medicine in Clinical Areas* was published [3] with 33 experts in each clinical area. In this paper, I aim to review the concept and necessity of SGM and the challenges and implications it presents to healthcare, practitioners, researchers, and students within the sex/gender-specific framework.

## Definition and necessity of sex/gender-specific medicine

SGM is important because even for the same disease, there are differences between men and women, and a different approach is necessary to identify the causes and provide treatment. These differences in SGM should be considered in two aspects: the sex aspect due to the differences in hormones or genes, and the gender aspect due to the difference in social and cultural roles between men and women. Although it is generally believed that there is no difference in the occurrence of diseases between men and women, the reality is that sex and gender shape both the occurrence and clinical course of diseases. SGM conducts research on these differences between men and women to achieve more accurate diagnosis and treatment directions, such as in bowel diseases where differences between men and women are particularly important.

Men and women often exhibit different symptoms of diseases and responses to treatment, which are caused by the effects of sex hormones or genetic predispositions, or by different sociocultural conditions that impact the diseases. In particular, gender considerations are crucial in gastrointestinal diseases such as functional dyspepsia and irritable bowel syndrome because these diseases are highly likely to occur due to stress, and the stress experienced by men and women is different due to gender differences.

It is natural to pay attention to the physical differences between men and women when dealing with the human body, and medicine has made progress by considering these differences. However, it is also true that the differences between men and women have not yet been adequately considered for most diseases’ causes or treatments. Fortunately, with the discovery of various sex/gender differences, research on sex/gender bias of disease occurrence is being actively conducted. Although most physicians in Korea are still unfamiliar with the term “sex/gender-specific medicine,” it is clear that the development of SGM will be an unstoppable trend from the macroscopic perspective of medical development.

## Why sex/gender-specific medicine appeared

Before the 1980s, every area of our society was male-centered, including the medical field. The medical field was male-centered enough to have the viewpoint that “a woman is a little man.” As a result, the diagnosis and treatment of most diseases were conducted with men at the center. Unfortunately, this bias led to the development of modern medicine that did not consider sex/gender differences, resulting in tragic cases. Here are some examples.

### Thalidomide

**Figure 1.**
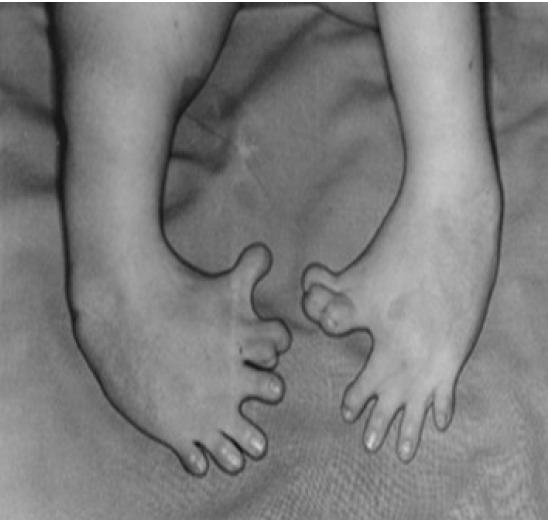
A baby with deformed toe after maternal thalidomide intake. Courtesy of Wikimedia Commons.

Thalidomide, which was called a miracle cure for morning sickness, received the most attention [4]. Thalidomide was first developed as a sedative in 1953 and marketed in 1957 under the brand name Contergan by Grünenthal Pharmaceuticals. Although clinical trials on humans were not conducted properly, adverse effects were rarely revealed in various animal experiments such as rats and rabbits. The drug was advertised as “a miracle drug with no adverse effects” and sold as a sedative and sleeping pill that could be purchased without a doctor’s prescription in about 50 countries around the world. Many pregnant women used it, particularly for soothing morning sickness. However, its danger was revealed when pregnant women who took this drug between 1960 and 1961 gave birth to deformed babies. Until sales were completely suspended in 1962, more than 10,000 deformed babies were born (Figure 1). Research on the drug revealed that taking it before the 42nd day of pregnancy resulted in the birth of a deformed baby with missing limbs, very short limbs, the complete absence of fingers or toes, or the absence of some fingers or toes due to the adverse effect of suppressing angiogenesis.

Later, during the process of developing new drugs, it was belatedly realized that humans, especially women and pregnant women, should be considered. Like the thalidomide case, after experiencing fatal adverse effects of drugs in women, women were excluded from clinical trials due to concerns about their fertility and unpredictable results. Researchers preferred to include only men in clinical trials in order to maintain other variables as consistent as possible except the drug. Women’s menstrual cycles and hormone changes were also considered factors that made clinical trials difficult. This trend has gradually changed since the 1990s, when the U.S. Food and Drug Administration (FDA) changed the research guidelines to include women in clinical trials [5]. However, there have been no significant changes in Korea yet.

### Cisapride

**Figure 2.**
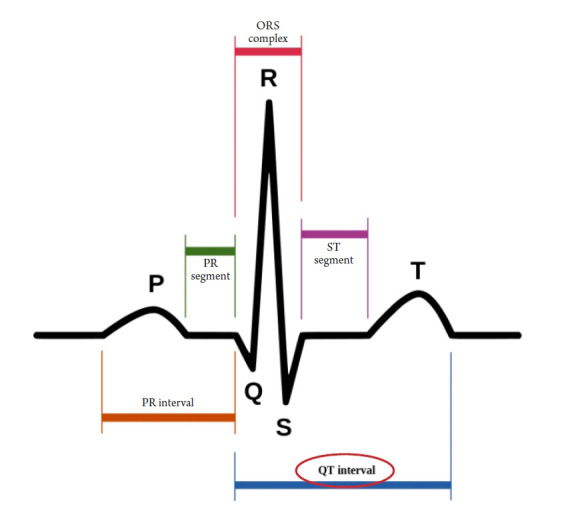
QT interval in normal echocardiogram. Courtesy of Wikimedia Commons.

The U.S. Government Accountability Office investigated 10 drugs withdrawn from the market between 1997 and 2021 due to adverse effects and found that eight of them showed a higher risk in women than in men. One reason for this is that women took those drugs more often, such as appetite suppressants. In some cases, physical differences between men and women caused more fatal adverse effects in women, such as with cisapride (brand name, Prepulsid; Janssen-Ortho). Cisapride is a drug that stimulates gastrointestinal motility by acting on serotonin receptors. It was first developed in 1980 and has been widely used to treat gastrointestinal diseases, including reflux esophagitis. As a gastroenterologist, I prescribed the drug frequently. In 2000, it was shocking to learn that a 15-year-old Canadian girl named Vanessa Young, who was prescribed the drug for stomach discomfort due to bulimia, had died of a heart attack. It was later discovered that cisapride could cause fatal cardiac arrhythmia, and the pharmaceutical company voluntarily stopped selling it [6]. This was a major surprise for gastroenterologists who prescribed the drug frequently. The QT interval, the length between the Q and T waves in an electrocardiogram, tends to be longer in women than in men. Cisapride made the QT interval longer, causing fatal arrhythmia and cardiac arrest, especially in women (Figure 2).

### Zolpidem

Zolpidem, a sleeping medication, has recently become a concern. In 2011, Lindsey Schweigert woke up in the back seat of a police car wearing her nightgown, but she could not remember why she was there, no matter how hard she tried. Later, it was discovered that this was due to symptoms such as sleepwalking as an adverse effect of zolpidem [7]. About 700 car accidents related to zolpidem were reported in the United States alone. It was confirmed that the rate of metabolism and excretion in the body after taking the drug is slower in women than in men, and a higher blood drug concentration is maintained in women than in men. In 2013, the FDA recommended that people taking zolpidem avoid driving or work that requires concentration the day after taking it. It was also recommended to lower the first prescription dose to 5 mg, half the previous dose, for women [8]. This case may also have occurred due to not considering the differences between men and women in the initial process of developing the drug and determining the dose.

Why are the adverse effects of drugs different between men and women? These discrepancies are primarily due to physiological differences. Zolpidem is absorbed well by fat. Since women have more body fat than men, the drug remains in women’s bodies longer. In addition, since men’s and women’s heart rates differ, cisapride may cause more fatal cases of arrhythmia in women. Changes in female hormones can also affect drug metabolism through the liver, and the smaller size of a woman’s kidney slows down the excretion rate of drugs. Since women have smaller body weight and surface area, the same dose of a drug may have a greater effect on them than on men. In addition to these physical differences, women tend to complain of chronic symptoms more than men, and therefore take more drugs, which exposes them more frequently to interactions among drugs. Therefore, it is necessary to consider sex/gender differences when developing drugs or using existing drugs.

## History of sex/gender-specific medicine

In the late 1980s, concerns about sex/gender bias in clinical trials and drug development were raised, prompting the U.S. National Institutes of Health (NIH) to announce a principle that women must be included in clinical trials [9]. The NIH’s policy was proposed as a law in 1993, requiring the inclusion of women and ethnic minorities in all human research. In phase 3 clinical trials, it was recommended to include sufficient numbers of women and ethnic minorities for subgroup analysis in the verification process of the treatment effect and to prohibit the exclusion of subjects due to cost [10]. At that time, the importance of SGM, which had previously been neglected, received attention and was recommended as a policy, but those recommendations were often not reflected in actual clinical trials [11,12]. However, starting about 10 years ago, SGM received attention in earnest, as major research grant institutions implemented regulations requiring SGM to be considered clinical trials [12]. Since funding is essential to conduct research, these regulations seem to be the most effective way to ensure that SGM is sufficiently addressed. The Canadian Institutes of Health Research (CIHR) since 2010 [13], the European Commission since 2014 [14], and the NIH since 2015 [10] have required that research grant applications must stipulate whether the research considers sex or gender and sex as a biological variable in research design, analysis, and reporting processes [9]. After GISTeR of KOFWST persuaded the National Research Foundation of Korea (NRF) and the Ministry of Health and Welfare (MOHW) that extra points should be given to research on sex/gender, NRF and MOHW research grants have taken a cautious approach, such as awarding extra points for female principal investigators and the inclusion of appended documents related to SGM. Along with the regulations on research grants, academic journals also play a significant role in promoting SGM. In 2014, the editors-in-chief of major biomedical journals gathered to discuss the problem that even research published in influential academic journals could not be replicated in follow-up experiments, and they pointed out that the insufficient consideration of sex/gender in the experiment and reporting processes was a major factor contributing to this problem [9].

Many academic journals have subsequently made it clear that they will consider the application of SGM in preclinical and clinical trials when reviewing articles, which has led researchers to pay more attention to the importance of SGM [15]. Numerous editors have become sympathetic to the fact that many published research articles do not even mention “male” or “female” in animal experiments or present separate analyses for men and women. In November 2017, KOFWST hosted a lecture entitled “Gender Application Cases in International Biomedical and Health Journals,” which introduced SGM to the editors-in-chief of biomedical journals in Korea, leading to the inclusion of content on SGM in the editorial policy of each journal [9]. Over the years, efforts to introduce SGM in biomedical research have led to the inclusion of content related to SGM in the ARRIVE (Animal Research: Reporting In Vivo Experiments) guidelines and recommendations of the International Committee of Medical Journal Editors, as well as the publication of SGM guidelines such as the SAGER (Sex and Gender Equity in Research) guidelines [16-18]. Additionally, information on SGM is readily available through online resources such as the Gendered Innovations Center at Stanford University and the Institute of Gender and Health under CIHR [9]. Progress in this area is steady.

## Examples of sex/gender-specific medicine

Research on SGM has revealed that there are significant differences between men and women in various medical areas such as myocardial infarction, heart failure, autoimmune diseases, depression, thyroid diseases, and diabetes. Applying these differences to medical treatment has led to significant outcomes. For example, the main symptom of myocardial infarction is chest pain in men, while women experience heartburn and chest tightness as the primary symptoms. Prior to SGM, only chest pain was recognized as the primary symptom, resulting in misdiagnosis and delayed treatment for women presenting with heartburn and chest tightness. However, SGM has led to proper identification of these symptoms and improved medical outcomes. Additionally, it was discovered that the treadmill test was less accurate for women, but was still performed equally on both men and women [19]. The advent of SGM led to the recognition that the treadmill test should be performed differently for men and women to reduce errors. These examples demonstrate the necessity of SGM in achieving better medical outcomes.

## The difference between sex and gender

**Figure 3.**
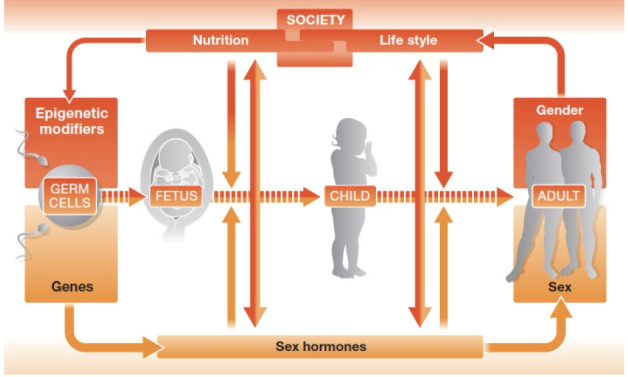
How sex and gender are closely interrelated in determining health. Adapted from Regitz-Zagrosek [20] with a permission from EMBO Press.

The differences between men and women in terms of hormones and genes are the main reasons why they show differences in diseases (Figure 3) [20]. Hormone differences are widely known, but genetic differences are not as well-known. Recent research has revealed that genetic differences between men and women are around 1%; this may seem like a small difference, but it is actually quite substantial, since the genetic difference between humans and chimpanzees is only 1.2%. These differences in hormones and genetics can contribute to differences in diseases between men and women. Other biological differences to consider are reproductive function and sex hormone concentrations, as well as the fact that women tend to have a higher percentage of body fat than men. Moreover, social and cultural factors related to gender differences also have a major impact on the development of diseases. These factors can include differences in behavior, lifestyle, and social experiences. For instance, it is already known that there are substantial differences in thoughts and behaviors between men and women, and it has been discovered that these differences can be associated with the development of certain diseases [21].

However, biological differences and gender differences are so closely related that it can be difficult to determine which of them have a greater influence on the development of diseases. For instance, men tend to show aggression due to their vigorous secretion of testosterone. This trait leads to aggressive behaviors and creates an environment that is more susceptible to health risks, indicating that a biological trait can influence gender. In such cases, it is not clear whether biological or gender impacts should be prioritized. Conversely, gendered behaviors can alter biological factors. For example, poor lifestyle habits such as alcohol consumption or excessive stress can cause genetic mutations in adults, children, and even fetuses. One clear fact is that although biological differences are crucial when young, gender differences become even more critical as individuals age. Therefore, more research should be conducted in the future, and the development of SGM will be determined accordingly. These efforts will undoubtedly aid in disease treatments and significantly impact healthy life expectancy.

## Concluding remarks

Although the field of SGM is expanding and more researchers, clinicians, and students are recognizing it as a component of personalized medicine, it has not yet been fully developed as a field of study. Unfortunately, many people still misunderstand SGM as research related to the women’s movement or sexual minorities. The reason for the emergence of SGM is to ultimately improve patient treatment outcomes. However, research in this field is still in its early stages, and Korea is in its infancy. To accurately identify differences in diseases between men and women, more scientists in medicine, nursing, and related health fields need to take an interest in researching and studying them. Moreover, continuing attempts to apply and promote SGM among healthcare practitioners, researchers, and students will help propel it forward in earnest.

## References

[1] Kim N (2021). Sex/gender-specific medicine in the gastrointestinal diseases.

[2] Kim N (2022). Sex/gender-specific medicine in the gastrointestinal diseases.

[3] Kim N (2022). Sex/gender-specific medicine in clinical areas.

[4] Vargesson N (2015). Thalidomide-induced teratogenesis: history and mechanisms. Birth Defects Res C Embryo Today.

[5] U.S. Food and Drug Administration (FDA) (1997). Food and Drug Administration Modernization Act (FDAMA) of 1997 [Internet]. https://www.congress.gov/105/plaws/publ115/PLAW-105publ115.pdf.

[6] Arnott W (2001). Cisapride and the Vanessa Young inquest. CMAJ.

[7] Pirestani J (2017). Ambien: does this popular sleep medication turn people into zombies? [Internet]. https://www.pharmacytimes.com/view/ambien-does-this-popular-sleep-medication-turn-people-into-zombies.

[8] Food and Drug Administration (FDA) (2018). FDA Drug Safety Communications. Risk of next-morning impairment after use of insomnia drugs; FDA requires lower recommended doses for certain drugs containing zolpidem (Ambien, Ambien CR, Edluar, and Zolpimist) [Internet]. https://www.fda.gov/files/drugs/published/Drug-Safety-Communication--Risk-of-next-morning-impairment-after-use-of-insomnia-drugs--FDA-requires-lower-recommended-doses-for-certain-drugs-containing-zolpidem-%28Ambien--Ambien-CR--Edluar--and-Zolpimist%29.pdf.

[9] Lee MY, Kim EJ, Shin A, Kim YS (2021). How to study the sex and gender effect in biomedical research?. Korean J Gastroenterol.

[10] National Institutes of Health (NIH) (2015). Consideration of sex as a biological variable in NIH-funded research [Internet]. https://orwh.od.nih.gov/sex-gender/nih-policy-sex-biological-variable.

[11] Johnson SM, Karvonen CA, Phelps CL, Nader S, Sanborn BM (2003). Assessment of analysis by gender in the Cochrane reviews as related to treatment of cardiovascular disease. J Womens Health (Larchmt).

[12] Legato MJ, Johnson PA, Manson JE (2016). Consideration of sex differences in medicine to improve health care and patient outcomes. JAMA.

[13] Johnson JL, Beaudet A (2012). Sex and gender reporting in health research: why Canada should be a leader. Can J Public Health.

[14] European Commission (2016). H2020 programme: guidance on gender equality in Horizon 2020, version 2.0 [Internet]. https://eige.europa.eu/sites/default/files/h2020-hi-guide-gender_en.pdf.

[15] McNutt M (2014). Journals unite for reproducibility. Science.

[16] Heidari S, Babor TF, De Castro P, Tort S, Curno M (2016). Sex and gender equity in research: rationale for the SAGER guidelines and recommended use. Res Integr Peer Rev.

[17] Kilkenny C, Browne WJ, Cuthill IC, Emerson M, Altman DG (2010). Improving bioscience research reporting: the ARRIVE guidelines for reporting animal research. PLoS Biol.

[18] International Committee of Medical Journal Editors (ICMJE) (2019). Recommendations for the conduct, reporting, editing, and publication of scholarly work in medical journals [Internet]. http://www.icmje.org/icmje-recommendations.pdf.

[19] Cremer PC, Wu Y, Ahmed HM, Pierson LM, Brennan DM, Al-Mallah MH (2017). Use of sex-specific clinical and exercise risk scores to identify patients at increased risk for all-cause mortality. JAMA Cardiol.

[20] Regitz-Zagrosek V (2012). Sex and gender differences in health. Science & Society Series on Sex and Science. EMBO Rep.

[21] Holingue C, Budavari AC, Rodriguez KM, Zisman CR, Windheim G, Fallin MD (2020). Sex differences in the gut-brain axis: implications for mental health. Curr Psychiatry Rep.

